# Provider Adherence to National Guidelines for Managing Hypertension in African Americans

**DOI:** 10.1155/2015/498074

**Published:** 2015-10-13

**Authors:** Jeanette Sessoms, Kathryn Reid, Ishan Williams, Ivora Hinton

**Affiliations:** University of Virginia School of Nursing, McLeod Hall, P.O. Box 800782, Charlottesville, VA 22908-0782, USA

## Abstract

*Purpose.* To evaluate provider adherence to national guidelines for the treatment of hypertension in African Americans.* Design.* A descriptive, preexperimental, quantitative method.* Methods.* Electronic medical records were reviewed and data were obtained from 62 charts. Clinical data collected included blood pressure readings, medications prescribed, laboratory studies, lifestyle modification, referral to hypertension specialist, and follow-up care.* Findings.* Overall provider adherence was 75%. Weight loss, sodium restriction, and physical activity recommendations were documented on 82.3% of patients. DASH diet and alcohol consumption were documented in 6.5% of participants. Follow-up was documented in 96.6% of the patients with controlled blood pressure and 9.1% in patients with uncontrolled blood pressure. Adherence in prescribing ACEIs in patients with a comorbidity of DM was documented in 70% of participants. Microalbumin levels were ordered in 15.2% of participants. Laboratory adherence prior to prescribing medications was documented in 0% of the patients and biannual routine labs were documented in 65% of participants.* Conclusion.* Provider adherence overall was moderate. Despite moderate provider adherence, BP outcomes and provider adherence were not related. Contributing factors that may explain this lack of correlation include patient barriers such as nonadherence to medication and lifestyle modification recommendations and lack of adequate follow-up. Further research is warranted.

## 1. Introduction

Hypertension (HTN) is a medical condition that is characterized by high or uncontrolled blood pressure. Inadequate control of HTN can lead to more serious vascular conditions affecting the major blood vessels in the heart, brain, and body. Additionally, HTN and diabetes mellitus (DM) frequently coexist, which further increases the risk of developing vascular complications. Vascular complications are a group of disorders that affects the heart and blood vessels. Hypertension is a major risk factor for vascular disease including heart attacks and strokes [1]. In 2008, an estimated 17.3 million people died from vascular complications. Of those 17.3 million vascular-associated deaths, 6.2 million were due to strokes [2]. It is predicted that, by the year 2030, an estimated 23.3 million will die from stroke and heart disease [2]. Addressing risk factors that contribute to HTN may help prevent vascular complications. According to the World Health Organization (WHO) [3], complications of HTN such as strokes account for 9.4 million of the astounding 17 million vascular-associated deaths. Another consideration is the financial burden of HTN; according to the Centers for Disease Control and Prevention (CDC) [4], the annual cost of HTN treatment was 131 billion dollars.

The physical and financial burdens of HTN are not unique to any one group of individuals. However, it has been well documented that African Americans (AAs) have a disproportionate burden of morbidity and mortality compared to Caucasians [1]. Data collected from 2008 suggest that non-Hispanic blacks accounted for 31.7% of the 59.4 million people with HTN, whereas non-Hispanic whites accounted for only 26.8% [2]. Despite research and interventions to decrease both the physical and financial burdens of uncontrolled HTN, specifically in the AA population, HTN remains a national problem [5].

Numerous interventions have been documented to improve control of HTN in AAs. The aims of such interventions have been to reduce the barriers to better control. Provider-centered barriers are the focus of this study and include limited patient-provider communication regarding lifestyle changes, lack of adherence to established guidelines for HTN management, and resistance to change. In addition, systems barriers were assessed and include access to care, medication costs, and lack of healthcare coverage [6]. Racial disparities related to geographical areas in healthcare lead to disproportionate mortality and morbidity in rural areas.

Patients often seek medical attention for chronic conditions from their primary care providers. Geographic location of this population and clinic locations can influence patient outcomes [7]. Rurality adds to the burden of HTN in AAs. Healthcare disparities such as ethnicity, poverty, and access to care are all associated with rurality and contribute to the higher incidence of HTN in AAs. For example, barriers to healthcare in rural communities include transportation, lack of health insurance, and lack of healthcare facilities and providers, all of which contribute to limited access to healthcare. As a result, rural communities have a higher incidence of chronic diseases such as HTN [7] and have poorer outcomes [8].

As previously mentioned, a major problem for rural communities is access to healthcare. Improving access to healthcare for rural America is a priority. The National Rural Health Association [9] has developed a timeline for the Affordable Care Act, which is designed to address the issues pertaining to access to healthcare. Provisions on the timeline include workforce improvement, payment reimbursement, and requirement of the electronic health record requirements, to name a few. Student loan repayment programs for those working in rural or underserved areas and improving Medicare and Medicaid reimbursement in rural practices are some specific provisions that have been implemented to improve access to healthcare in rural communities [9].

## 2. Methods

### 2.1. Theoretical Framework

The theoretical framework of Avedis Donabedian was used as a tool to guide this research. His framework was used to assess the quality of care provided in healthcare. The three components that form the foundation of this theory are (1) structure of care, (2) process of care, and (3) outcomes. The concept is grounded on the principle of healthcare outcomes as a result of the medical care provided by medical professionals [10].

**Figure 1:**
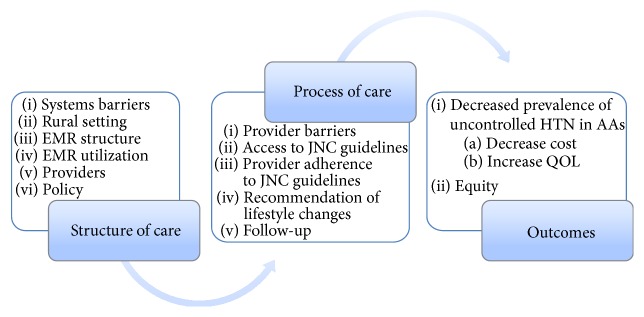
Application of Donabedian's quality of care.

Donabedian (as cited in McDonald et al. [11]) describes structure of care as any process that relates to the organizational and physical aspects of care settings. A few specific examples of this process are facilities, equipment, and operational and financial processes supporting medical care. The second component of this framework is process of care. Process of care is dependent upon the structures of care to supply resources and methods that are necessary for participants to carry out patient care activities. Patient-provider communication, practice habits, and care management are all examples of process of care. Further, the goal of process of care is to improve patient health by promoting recovery, patient survival, and even patient satisfaction [10]. The final concept of this model, outcomes, is simply the patient outcomes based on medical health after the application of the two previous components [10].  Figure 1 depicts the components of Donabedian's theory and how it is applicable to this study.

### 2.2. Study Design

**Figure 2:**
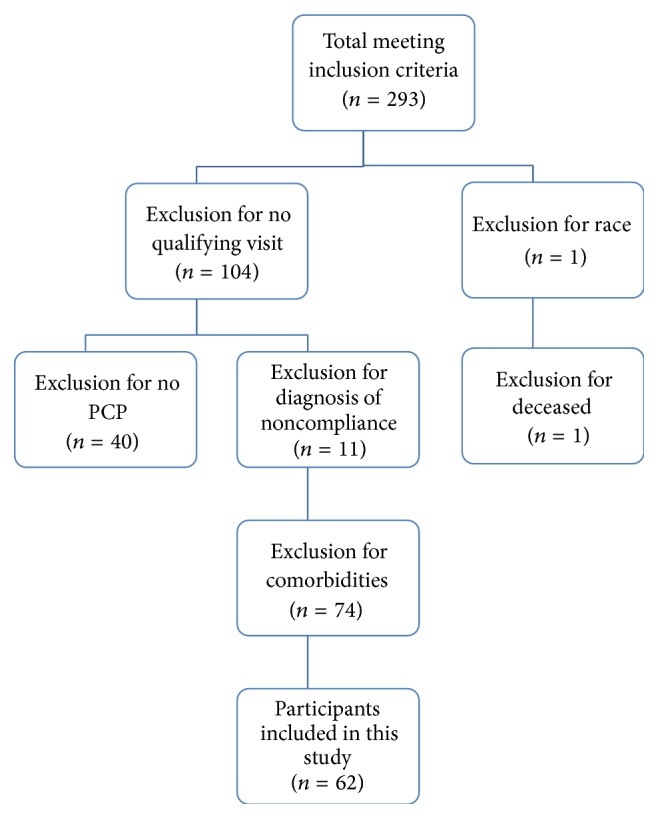
Participant selection algorithm.

A retrospective review of the EMR was conducted to identify hypertensive AA patients in a rural clinic who were seen from July 1, 2014, to August 31, 2014. A descriptive, preexperimental, quantitative method was used to evaluate the degree of provider adherence to national HTN guidelines in AAs living in a rural community. Inclusion criteria for the patients included (Figure 2) (a) age 20 to 80 years, (b) AAs with a diagnosis of HTN, and (c) receiving antihypertensive medications. Exclusion criteria included (a) specific end organ damage (i.e., CKD, stroke, cardiomyopathy, or myocardial infarctions), (b) age younger than 20 or greater than 80 years, (c) no office visits during research dates or office visits for reasons other than HTN, (d) no established relationship with a single primary care provider (PCP), (e) diagnosis of medical nonadherence, (f) race other than AA, and (g) deceased patients.

A sample of 62 participants met the inclusion criteria.

### 2.3. Study Setting and Sample

The study was conducted at a multiphysician practice located in a rural community. The practice serves a population of 45,273, 63.8% of which are AAs [12]. The practice accepts Medicare, Medicaid, private insurances, and the indigent. The practice serves pediatric to geriatric patients. There are four primary care providers, one cardiologist, two pulmonologists, one neurologist, and one podiatrist. Primary care providers were the focus of this study. The study aims to assess healthcare provider adherence to JNC hypertensive guidelines in AAs.

### 2.4. Data Collection and Procedures

The primary source data were selected from the EMR Centricity developed by General Electric Healthcare. An EMR is a digital or electronic version of a paper chart that contains the patient's medical history. A report was populated using the following criteria: (1) the practice site location, (2) race specified as black or African American, (3) birthdate on or after 01/01/1949 but before 01/01/1995, (4) appointment date on or after 07/01/2014 but before 09/01/2014, and (5) active International Classification of Diseases, Ninth Revision (ICD-9) codes containing 401 for hypertension. The EMR was reviewed to identify onset of HTN if feasible, provider selection of antihypertensive drugs for initial treatment, and additional drug choices. HTN was defined as a blood pressure ≥140/90 mmHg in the general population and >130/80 mmHg in hypertensive individuals with a comorbidity of DM in accordance with JNC 7 or patients taking antihypertensive medications. Patients who met the inclusion criteria were selected by convenience sample. A list of those patients was composed and each was assigned a research identifier. Potential participants were consecutively recruited and a sample size of 62 participants met the inclusion criteria. Demographic variables assessed included age, gender, marital status, and insurance coverage. Other variables that were considered include the following antihypertensive drug classes: (a) thiazide diuretics, (b) angiotensin-converting-enzyme inhibitors (ACEIs), (c) angiotensin II receptor blockers (ARBs), and (d) calcium channel blockers (CCBs). Evaluation of monotherapy and combination therapy was also performed.

### 2.5. Measures

JNC 7 (as cited in Chobanian et al. [13]) describes HTN as a systolic blood pressure ≥140 mmHg or a diastolic blood pressure of ≥90 mmHg in the general population, including AAs. If the patient has a comorbidity such as DM, a systolic blood pressure >130 mmHg and a diastolic blood pressure of >80 mmHg are considered suboptimal in the treatment of HTN. The coexistence of HTN and DM further increases the risk of vascular complications such as strokes and renal disease, which is why the optimal blood pressure goal is lower [13].

Diagnostic measurements for the classification of HTN were performed based on JNC 7 guidelines (Table 1). According to JNC 7, two consecutive readings in contralateral arms at least 5 minutes apart while sitting are categorized as HTN. By auscultation, blood pressures were manually obtained by nurses using the appropriate size cuff with a sphygmomanometer. Patients were in a seated position with feet on the floor and arm positioned at the level of the heart. They had been seated for at least 5 minutes or longer. The providers performed repeat BPs in suboptimal readings after at least 5 minutes. JNC 7 describes the stages of HTN. A normal blood pressure is a systolic blood pressure of <140 mmHg and a diastolic blood pressure of <90 mmHg. Stage 1 is a systolic blood pressure reading of 140 to 159 mmHg or a diastolic BP reading of 90 to 99 mmHg. Stage 2 is classified as a systolic blood pressure ≥160 mmHg and a diastolic reading of ≥100 mmHg. In the general AA population, initial monotherapy with diuretics, specifically thiazide diuretics (TDs), or CCBs should be used for stage 1 HTN or a diuretic in combination with other drug classes for combination drug therapy regimen for stage 2 HTN. JNC 7 recommends specialty referrals if blood pressure is not controlled after maximizing three medication classes, with one being a TD. Lastly, for those with compelling indications, such as those with a comorbidity of DM, ACEIs are recommended to reduce strokes and other vascular complications [13].

With regard to follow-up, JNC 7 recommends a monthly follow-up office visit if blood pressure is not at goal and a follow-up office visit every 3 to 6 months if BP is at goal. Laboratory values for potassium and creatinine should be obtained 1 to 2 times annually and patients with a comorbidity of DM should have their urine microalbumin levels measured at least annually. Patients newly diagnosed with HTN should have a urinalysis, blood glucose, hematocrit, potassium, creatinine, calcium, and lipid profile drawn prior to beginning pharmacological treatment.

JNC 7 recommends lifestyle modification education. Better outcomes have been found when lifestyle modification is incorporated into the plan of care. The following are the areas recommended for lifestyle modification: (a) weight loss, (b) following the Dietary Approaches to Stop Hypertension (DASH) diet, which consists of a diet rich in fruit and vegetables, low fat dairy products, and reduced intake of saturated and total fat, (c) adhering to sodium restrictions, (d) regular physical activity, and (e) limiting alcohol consumption.

### 2.6. Data Analysis

Statistical analyses were performed on the outcomes of blood pressure control in participants who were prescribed antihypertensive medications based on JNC 7 guidelines compared to those who were not and also on blood pressures that were at goal and those that were not. Additionally, outcomes of provider adherence to the guidelines were measured based on adherence to medication choice recommendations, documented lifestyle modification recommendations, laboratory studies, and follow-up for patients with HTN and HTN with a comorbidity of DM. Descriptive analysis was conducted using crosstabs, frequencies, and means comparison and reported as percentages to describe the results. Crosstabs were used to determine the number of times the recommended combination of a thiazide diuretic was used in combination with an ACEI or ARB. Frequencies were conducted to identify the percentage of patients not prescribed a TD or CCB as monotherapy. Further, the use of means comparison was to compare differences in BP outcomes in patients prescribed ACEIs compared to TDs as monotherapy. Additionally, a chi square analysis was performed to determine if there is a relationship between provider adherence and blood pressure outcomes.

## 3. Results

### 3.1. Providers

Physicians accounted for 64.5% (*n* = 40) of the providers in this study. Nurse practitioners (NPs) accounted for 35.5% (*n* = 22). Of the 29 patients with blood pressure at goal, a physician was the provider in 75.9% (*n* = 22) of the office visits while NPs provided care in 24.1% (*n* = 7) of the visits.

The demographic characteristics of the 62 participants are described in Table 2. Of the 62 patients studied, 41.9% were male and 58.1% were female. Patient age was divided into 2 categories: less than 65 years and 65 years and older. There were 50% patients in each age group. The mean age was 62.8 years. The most frequent stage of uncontrolled HTN was stage 1 accounting for 84.4% of the 32 patients. Stage 2 HTN was detected in 15.6% of the patients. There were 16 nondiabetic patients whose BP was not at goal. Of those 16, 81% had stage 1 HTN and the remaining 19% had stage 2 HTN. In patients aged less than 65 years, 45.2% of the 31 patients had stage 1 HTN and 12.9% had stage 2 HTN. In the age group of 65 and over, stage 1 accounted for 41.9% of patients and stage 2 HTN accounted for 3.2%. Medicare coverage accounted for 56.5% of the study participants. Blue Cross Blue Shield (BCBS) accounted for the second most utilized health insurance with 24.2% of the patients enrolled. Medicaid, self-pay, and private insurances each accounted for 6.5% of the patients.

### 3.2. Pharmacologic Treatment

Drug therapy regimens are reported in Table 3. Of the 62 patients studied, 12.9% (*n* = 8) were on monotherapy and 87.1% (*n* = 54) were on combination therapy. Combination therapy is described as taking two or more medications. Those with a comorbidity of DM accounted for 53.2% (*n* = 33) of the 62 patients. JNC 7 guidelines suggest that patients with a comorbidity of DM should take ACEIs to decrease morbidity and mortality. Of the 33 diabetic patients, only 69.7% (*n* = 23) were taking ACEIs as recommended. TDs or CCBs were not prescribed in any of the eight patients on monotherapy. Of the 54 patients taking combination therapy, 87% (*n* = 47) were taking either a TD or CCB. In the patients studied, 53.2% (*n* = 33) of the 62 warranted medication adjustments as a result of uncontrolled blood pressure. Only 15.2% (*n* = 5) had medication adjustments, leaving 84.8% (*n* = 28) inadequately treated. One patient (1.6%) required a referral to a HTN specialist as a result of maximizing three different medication classes, including a TD. That patient was not referred.

In the eight patients on monotherapy, 37.5% (*n* = 3) of them met their blood pressure goal despite not being on a TD or CCB. Of the 54 patients on combination therapy, 87% (*n* = 47) were on a TD or CCB as recommended by JNC 7. Of those 47 patients, 55.3% (*n* = 26) achieved goal blood pressures. A chi square test was used to determine if there is a relationship between being prescribed JNC 7 medication regimen and blood pressure outcomes. There was no significant relation between taking the recommended TD or CCB and blood pressure control (*χ* ^2^ = 0.0; *P* = 0.99).

### 3.3. Lifestyle Modifications

**Figure 3:**
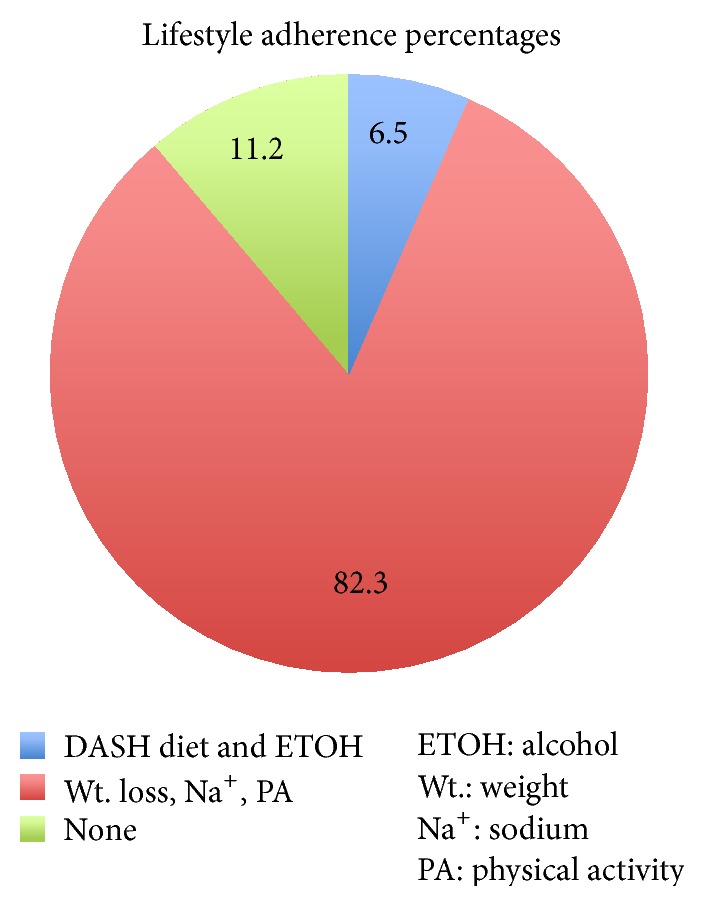
Lifestyle adherence.

The categories examined under lifestyle modifications included DASH diet, weight loss, sodium restrictions, physical activity (PA), and alcohol consumption (see Figure 3). Only 6.5% (*n* = 4) of the 62 patients had documentation of provider recommendations for the DASH diet and alcohol consumption. The DASH diet includes recommendations for limiting alcohol consumption. Weight loss, sodium restriction, and PA recommendations were documented in 82.3% (*n* = 51) of the patients.

### 3.4. Follow-Up Care

JNC 7 recommends follow-up every 3 to 6 months if BP is controlled. Providers were adherent to follow-up recommendations 96.6% (*n* = 28) of the time in the 29 patients with controlled blood pressure. In the remaining 33 patients who required monthly follow-up due to uncontrolled blood pressure, providers were only 9.1% (*n* = 3) adherent to the recommendations.

### 3.5. Laboratory Recommendations

Providers were adherent to obtaining laboratory tests prior to initiating treatment in 0% of the two patients with new diagnoses of HTN. The adherence rate for biannual laboratory tests in patients with historical diagnosis of HTN was 65% (*n* = 39) of the 60 qualifying patients. In patients with a comorbidity of DM, JNC 7 recommends measuring annual urine microalbumin levels. Providers were 15.2% (*n* = 5) adherent to the guidelines in the 33 diabetic patients.

## 4. Discussion

Endless and organized activities that result in measurable improvement in healthcare services and targeted patient outcomes have been described as QI [14]. The way care is delivered is related to quality. The US Department of Health and Human Services [14] identified the 4 principles of quality improvement (QI) as (1) QI work as systems and processes, (2) focus on patients, (3) focus on being part of the team, and (4) focus on use of data. QI work as systems and processes refers to resources and activities that are carried out and are evaluated simultaneously to improve quality of care or outcomes [14]. This is modeled after Donabedian's framework for quality improvement. This study focused on the systems or structural components of systems barriers such as the rural setting the study was conducted in, EMR structure, EMR utilization, providers, and policy. Activities assessed included provider barriers, access to JNC 7 guidelines, provider adherence to those guidelines, recommendation of lifestyle changes, laboratory assessment, and follow-up.

Evaluation of the first 2 components is necessary to produce or improve patient outcomes. Outcome goals include decreasing the prevalence of uncontrolled HTN in AAs, decreasing cost associated with HTN, increasing quality of life, and equity. This study was conducted to assess what is currently being done in this rural primary care setting to address the increased prevalence and mortality of HTN in AAs. Using the methodical framework of Donabedian, both quantitative (frequencies) and qualitative (descriptive) data were collected and analyzed to assess the current system and to identify areas for improvement. The practice guidelines of JNC 7 were used as performance measures for comparison.

The JNC conducts and analyzes evidence-based studies periodically. Subsequently, JNC formulates recommendations based on those findings. The National Heart Lung and Blood Institute (NHLBI) traditionally has endorsed previous versions. Controversy surrounding the Eighth Report of the Joint National Committee (JNC 8) has led to NHLBI not endorsing JNC 8 [15]. Other federal organizations have also declined to endorse the new recommendations. The controversy surrounds the increased BP goal of <150/90 in patients aged >60 and a goal of <140/90 for those aged 18 to 60, including those having comorbidities of DM and CKD. JNC 8 guidelines were avoided for this study due to this controversy and its relatively new release.

There are several main findings of this study. First, the first-line drug choice as monotherapy in the treatment of HTN in AAs should be TDs or CCBs, as recommended by JNC 7. While there were only eight patients receiving monotherapy, none of them were on TDs or CCBs, which indicates 100% nonadherence to the guidelines regarding monotherapy. In fact, the majority of the patients on monotherapy were on ACEIs, while the remaining were on ARBs. However, studies have found that ACEIs and ARBs are less effective in the AA population. These findings are consistent with the studies reviewed in the literature review for this study. One such study compared the effectiveness of ACEIs as monotherapy between AAs and Whites [16]. Whites had a greater systolic (mean difference of 4.64) and diastolic (mean difference of 2.82) reduction in BP compared to AAs. In contrast, providers were more consistent with the guideline recommendations in AAs on combination drug therapy. Provider adherence was documented in 87% of the patients receiving combination therapy. This finding is consistent with previous studies. One of the goals of a previous study was to determine provider adherence to national guidelines including a TD in combination therapy in AAs of Nigerian decent [17]. The majority (88.8%) of the study sample was prescribed combination therapy inclusive of a TD. In addition, combination therapy was more effective than monotherapy in reducing both systolic and diastolic BP (32.64 mmHg compared to 15.43 mmHg and 18.56 mmHg compared to 6.96 mmHg, resp.).

Less than half of the 62 patients in this study have at goal BP readings at the level recommended by JNC 7 despite moderate provider adherence. Similar findings were found in a previous study. Provider adherence to the guidelines overall was 76%. Mean BP values decreased but insignificantly concluding no correlation with provider adherence and attaining BP goals [18]. In this study, medication adjustments were not made in 18% of the 33.2% that required adjustments. This could be a contributing factor to patients not meeting their BP goal. However, adjusting medications is not always associated with attaining BP goals [18]. Additionally, provider adherence in prescribing ACEIs in patients with a comorbidity of DM was seen in 70% of the population. Similar results were found in a previous study. Provider adherence to prescribing an ACEI or ARB in patients with comorbidities such as DM was seen in 88% of the population [18]. Studies have shown that the use of ACEIs in this population decreases mortality and morbidity by decreasing end organ damage and cardiovascular incidents.

Lifestyle modification, as an adjunct to pharmacologic therapy, has been associated with better BP control. Provider adherence to alcohol consumption and DASH diet recommendations was poorly documented. Detailed recommendations for the DASH diet and alcohol were only documented in 4 of the 62 EMRs. Adherence was high in the recommendations for physical activity, weight loss, and sodium restrictions. The smart plan is inclusive of these 3 recommendations, which was documented in 51 of the 62 patients.

Patient adherence with adequate office visit follow-up has been known to yield better BP control. JNC 7 recommends an office visit follow-up every 3 to 6 months in patients with BP at goal and monthly visits for those who are not at goal. Provider adherence was evident in the 3-to-6-month follow-up population (97%). A monthly follow-up recommendation for those with uncontrolled BP was poorly represented in this study.

Uncontrolled BP can lead to end organ damage such as renal insufficiency or failure, heart attacks, or strokes. Further, the medications used to treat HTN can have adverse effects on other organs. JNC 7 recommendations include a urinalysis, blood glucose, hematocrit, potassium, creatinine, calcium, and lipids in patients newly diagnosed with HTN before initiating therapy. Due to the limitations of the EMR, only 2 patients were identified as newly diagnosed. Of these patients, neither of them had laboratory testing performed prior to starting treatment. Diabetics should have their urine microalbumin level measured annually. Only 15% of diabetics had documented microalbumin levels within the preceding 6 months.

There are several limitations to this study. Due to small sample size, variability and vagueness should be noted as limitations. For example, the finding of no documentation of patients receiving labs prior to the initiation of drug therapy may provide a greater impact and consistency in a larger sample size. A larger sample size would produce more detailed, robust, and explanatory assessments. Secondly, the study was conducted during only the summer months and over a short duration. Extending the study period and expanding the study to include fall or winter months may provide input for comparison to determine if seasons impact BP control.

## 5. Conclusions

Despite evidence-based recommendations by JNC 7, provider adherence in AAs has room for improvement. Provider pharmacologic choices and lifestyle modification recommendations are major components to blood pressure control in this population. Thiazide diuretics are recommended as initial monotherapy and in combination therapy for African Americans. CCBs are recommended as an acceptable alternative to thiazide diuretics. CCBs are preferred over ACEIs because of the increased risk of stroke, myocardial infarctions, and other vascular conditions associated with ACEIs. Conversely, providers have demonstrated a preference in prescribing ACEIs and ARBS in monotherapy. Better adherence in prescribing a TD or CCB is seen in prescribing patterns for patients on combination therapy. Providers are not adherent to the monthly follow-up recommendations required for medication adjustment or specialist referrals when BP is not at goal. Lack of lifestyle modification documentation, specifically the DASH diet and alcohol consumption, is consistent with nonadherence to the JNC 7 guidelines. Although there appears to be no relationship between receiving the recommended medications and BP outcomes, more than half of the population did not meet BP goals.

The principal factor assessed in the process of care was provider barriers. Specific components impacting provider barriers include access to JNC 7 guidelines, provider adherence to JNC 7 guidelines, recommendation of lifestyle changes, and follow-up. Provider adherence to the guidelines overall was poor. Lack of documentation, provider-prescribing habits, and lack of knowledge of up-to-date, evidence-based guidelines may be contributing factors. While there is a gap between evidence-based national guidelines and clinical practice to controlling HTN, all contributing factors, including physician, patient, and systems barriers [19], require further exploration if successful interventions are to be developed.

## Figures and Tables

**Table 1:** Joint National Committee Classification of blood pressure for adults.

Blood pressure classification	Systolic blood pressure (mmHg)	Diastolic blood pressure (mmHg)
Normal	<120	and <80
Prehypertension	120–139	or 80–89
Hypertension	≥140	≥90
Stage 1 hypertension	140–159	or 90–99
Stage 2 hypertension	≥160	or ≥100

**Table 2:** Demographics.

Characteristic	Frequency	Percent
Gender		
Male	26	41.9
Female	36	58.1
Total	62	100.0

Age		
<65 years	31	50.0
≥65	31	50.0
Total	62	100.0

Stages of hypertension		
Stage 1	5	8.1
Stage 2	27	43.5
Controlled	30	48.4
Total	32	100.0

Marital status		
Married	19	30.6
Divorced	6	9.7
Single	14	22.6
Widowed	8	12.9
Undetermined	15	24.2
Total	62	100.0

Insurance		
Medicare	35	56.5
Medicaid	4	6.5
BCBS	15	24.2
Self-pay	4	6.5
Private	4	6.5
Total	62	100.0

**Table 3:** Medication regimen.

On TD or CCB	Frequency (*n*)	Percent
Monotherapy with TD or CCB (*n* = 8)		
Yes	0	0
No	8	100
Total	8	100.0

Combination therapy with TD or CCB (*n* = 54)		
Yes	47	87.0
No	7	13.0
Total	54	100.0

ACEI if there is comorbidity of DM (*n* = 33)		
Yes	23	69.7
No	10	30.3
Total	33	100.0

TD: thiazide diuretic; CCB: calcium channel blocker; ACEI: angiotensin converting enzyme inhibitor; DM: diabetes mellitus.
